# Supplementary material for: Splice-Junction-Based Mapping of Alternative Isoforms in the Human Proteome
Source: Cell Rep. Author manuscript; Available in PMC 2020 Jan 15. (PMC6961840; doi:10.1016/j.celrep.2019.11.026)
Supplement: 2 [file NIHMS1546469-supplement-2.zip › DF1/PXD009737/TestisTrypsin/TestisTrypsin_23_LGALS1_GGSLNLKPGECLR.pdf]

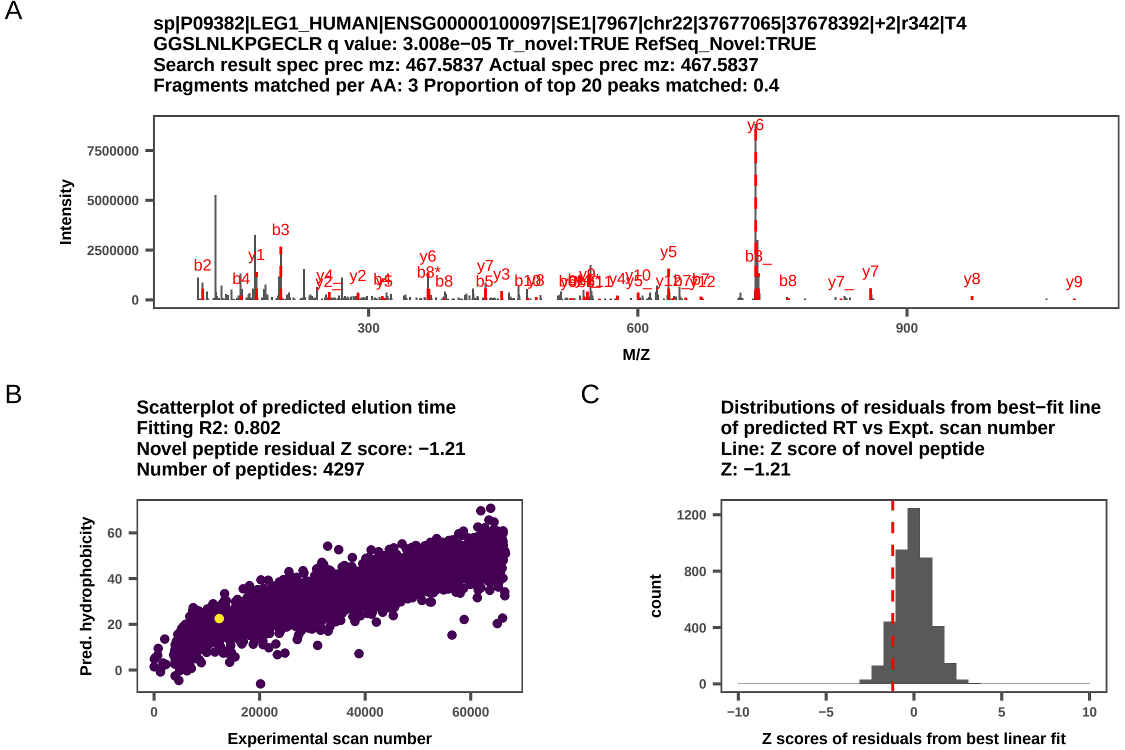
A
sp|P09382|LEG1_HUMAN|ENSG00000100097|SE1|7967|chr22|37677065|37678392|+2|r342|T4
GGSLNLKPGECLR q value: 3.008e−05 Tr_novel:TRUE RefSeq_Novel:TRUE
Search result spec prec mz: 467.5837 Actual spec prec mz: 467.5837
Fragments matched per AA: 3 Proportion of top 20 peaks matched: 0.4
Intensity
7500000
5000000
2500000
0
300
600
900
M/Z
y6
b3
b2
y1
b4
y4
y2
y6
b8*
b8
y7
b5
y3
y5
y10
b8_
b8
y7_
y7
y8
y9
B
Scatterplot of predicted elution time
Fitting R2: 0.802
Novel peptide residual Z score: −1.21
Number of peptides: 4297
Pred. hydrophobicity
60
40
20
0
0
20000
40000
60000
Experimental scan number
C
Distributions of residuals from best−fit line
of predicted RT vs Expt. scan number
Line: Z score of novel peptide
Z: −1.21
count
1200
800
400
0
−10
−5
0
5
10
Z scores of residuals from best linear fit
